# Outgrowing the Immaturity Myth: The Cost of Defending From Neonatal Infectious Disease

**DOI:** 10.3389/fimmu.2018.01077

**Published:** 2018-05-29

**Authors:** Danny Harbeson, Rym Ben-Othman, Nelly Amenyogbe, Tobias R. Kollmann

**Affiliations:** ^1^Department of Experimental Medicine, University of British Columbia, Vancouver, BC, Canada; ^2^Department of Pediatrics, Division of Infectious Diseases, University of British Columbia, Vancouver, BC, Canada

**Keywords:** neonate, infection, defense, tolerance, sepsis

## Abstract

Newborns suffer high rates of mortality due to infectious disease—this has been generally regarded to be the result of an “immature” immune system with a diminished disease-fighting capacity. However, the immaturity dogma fails to explain (i) greater pro-inflammatory responses than adults *in vivo* and (ii) the ability of neonates to survive a significantly higher blood pathogen burden than of adults. To reconcile the apparent contradiction of clinical susceptibility to disease and the host immune response findings when contrasting newborn to adult, it will be essential to capture the entirety of available host-defense strategies at the newborn’s disposal. Adults focus heavily on the disease resistance approach: pathogen reduction and elimination. Newborn hyperactive innate immunity, sensitivity to immunopathology, and the energetic requirements of growth and development (immune and energy costs), however, preclude them from having an adult-like resistance response. Instead, newborns also may avail themselves of disease tolerance (minimizing immunopathology without reducing pathogen load), as a disease tolerance approach provides a counterbalance to the dangers of a heightened innate immunity and has lower-associated immune costs. Further, disease tolerance allows for the establishment of a commensal bacterial community without mounting an unnecessarily dangerous immune resistance response. Since disease tolerance has its own associated costs (immune suppression leading to unchecked pathogen proliferation), it is the maintenance of homeostasis between disease tolerance and disease resistance that is critical to safe and effective defense against infections in early life. This paradigm is consistent with nearly all of the existing evidence.

## Introduction

The world has seen under-five mortality greatly reduced over the last two decades but this progress has least benefited those in the first 28 days of life—the neonatal period—which now accounts for nearly half of all under-five deaths (1). Infectious disease is one of the most common causes of newborn death, accounting for more than a third of all neonatal mortality (1). Unfortunately, the underlying reasons for this are not clear, preventing a rational approach to preventing newborn death across the globe. Unquestionably, newborns are much more susceptible to infection causing clinical disease (2–5). Also clear is that the neonatal immune system is very different than that of adults (6, 7). Many immunomodulatory approaches to improving outcome in neonatal infectious disease have been unsuccessful (8, 9), which necessitates a careful reexamination of our assumptions and beliefs regarding the nature of neonatal immune responses. The current dogmatic view, namely that the neonatal immune system is immature and therefore deficient to resist infection as compared to that of an adult (10), is inadequate as it does not capture the existing body of evidence (11). We recently reviewed the molecular mechanisms guiding the ontogeny of immune response from birth throughout infancy, emphasizing that newborns harbor an immune phenotype that is a match to the unique environmental pressures and challenges in the first days of life (11, 12). Balancing disease tolerance and resistance, while a challenge throughout the entire life span, is also unique for newborns (12). We here place the existing evidence in a larger framework to expand on this concept of host defense as a balance between disease tolerance and resistance to help guide the search for actionable answers.

## Neonatal Host Defense from Infectious Disease: The Complete Picture

Host defense to infection can broadly be divided into three different, not mutually exclusive, categories: disease avoidance, disease resistance, and disease tolerance (13). A more detailed exploration into the finer details of these defense strategies has been previously been outlined by Medzhitov et al. in their 2012 *Science* paper titled “Disease Tolerance as a Defense Strategy” (13). Here, each strategy is briefly summarized and connected explicitly to the neonatal immune response, which has been found time and time again to be distinct from the adult immune response (4, 11, 14–17):

(i) An avoidance strategy reduces the risk of infection by preventing exposure to infectious agents. Human avoidance of rotten meat consumption through an olfactory response to the metabolites produced by bacteria breaking down tissue is an example of avoidance (13). Limitations in both newborn mobility and exclusive breastfeeding can prevent potentially harmful exposure and represent an example of an avoidance strategy in early life (18). However, there are obvious physiological, physical as well as social and cultural limitations to this strategy; there is an unfortunate inevitability to some degree of pathogen exposure.

When avoidance has failed and infection has been established, the (ii) disease resistance approach aims to reduce pathogen burden and has traditionally been considered the primary *modus operandi* of the immune system (and thus the focus of most prophylactic or therapeutic interventions). However, unleashing antimicrobial immune responses can also cause collateral damage (13). In fact, much of what is clinically recognized as signs and symptoms of infection relates to this immune pathology (19). For example, a recent comparison of sepsis models showed that lipopolysaccharide (LPS) treatment in mice “induced a very similar course of inflammation” as infection (20). Given that LPS has no intrinsic virulence, the pathology of LPS challenge must result from host response, and thus similarities between LPS-induced sepsis and, e.g., polymicrobial sepsis (21) implicate host-mediated immune pathology as a key agent of disease. This is further evidenced by murine studies showing that knocking out anti-inflammatory cytokine production during infection is associated with worse outcomes without impacting bacterial clearance or viral replication (22, 23). Importantly, the newborn is particularly susceptible to this host-mediated immune pathology (e.g., intraperitoneal LPS challenge at 10 mg/kg resulted in 100% mortality in neonatal mice and 0% in adults) (11, 24). It is therefore not surprising that evolution has selected for a higher threshold that needs to be overcome in early life before a full-fledged immune response can be unleashed (12). This leaves the newborn with a conundrum; a disease avoidance approach has clear limitations [indiscriminately avoiding bacterial colonization is not only impossible, but would be harmful as the first few days of life are extremely important for establishing a healthy and diverse community of commensal enteric bacteria (25)], while a disease resistance approach carries substantial risk for immune-mediated damage (11, 13, 24, 26).

Newborns thus likely also rely on employing the third strategy of host defense, disease tolerance. (iii) Disease tolerance reduces potential harm to the host without reducing pathogen burden, generally by minimizing the level of immunopathology that results from a resistance response (13). This strategy is understood to be widely employed by plants (27) but the notion that animals (and humans) may rely on a disease tolerance defense as well has only recently begun to be considered (13). It is important to note that disease tolerance is different from the concept of adaptive immune tolerance: the former is a broad, categorical term for a defense strategy of coping with infection, and the latter is the immunological phenomenon of immune unresponsiveness to specific antigens. To our knowledge, the concept of disease tolerance as a defense strategy in early life has never been experimentally examined. However, existing evidence, while not proof, is at least consistent with its existence. Lastly, the host microbiota has increasingly been recognized as key to host defense, impacting all aspects of from avoidance (colonization resistance) to immune development; however, its role and relation to disease tolerance is significant and unexplored, as the tolerance to a range of microbial commensals is essential for a healthy human host (28).

## The Case for Higher Disease Tolerance in Early Life

A disease tolerant vs. intolerant phenotype would be expected to display a lower morbidity/mortality relative to a same pathogen load, and/or a higher pathogen load at a similar mortality level (13). While many suspected cases of bacterial sepsis in both neonates and adults are not confirmed by a positive blood culture (29, 30), within culture-positive cases, neonates have consistently been found to exhibit much higher circulating bacterial loads than adults (31). Despite expected variability depending on the pathogen involved, studies generally report bacterial counts in adults (with an active bacterial infection) to be somewhere in the range of 1–30 CFU/ml blood (31–34), while in neonates, the more commonly detected range lies between 50 and 500 CFU/ml blood with one-third of infected newborns harboring bacterial counts in excess of 1,000 CFU/ml (31, 35). Furthermore, while 50% of adult culture-positive cases harbor <1 CFU/ml blood (considered a “low” bacterial load), 78% of culture-proven newborn sepsis cases reported >5 CFU/ml of blood, and <50 CFU/ml blood was considered to be low for neonates (31). Most of these studies were not set up to compare newborn vs. adult bacterial loads in sepsis but rather were framed in the context of describing how much blood would be needed in order to confidently determine a culture-positive or a -negative state, thus do not directly address this comparison (34). However, this relationship also holds true in more controlled animal models, where much higher bacterial counts are consistently found in the blood as well as visceral organs of septic neonatal vs. adult mice challenged with the same pathogen (36).

Perhaps, a higher bacterial load in infected newborns does not seem surprising at first glance—after all, neonates are more susceptible to suffer from infection, and higher bacterial loads would seem to be entirely in line with this observation. However, this simple concept begins to unravel when age-specific mortality statistics are taken into consideration. While bacterial load correlates with outcome across all ages, there are log-fold differences in the scale of circulating bacteria which neonates are able to survive in comparison to adults. Studies have shown 100% mortality in adult patients with greater than 100 CFU/ml blood (37) and 84% mortality when the bacterial load was greater than 5 CFU/ml blood (38). By contrast, a cohort of neonates with sepsis suffered 73% mortality when bacterial loads were greater than 1,000 CFU/ml blood and 37% when less than 1,000 CFU/ml blood (35). This particular study describes the “low bacterial count with 37% survival” group as those with bacterial loads between 5 and 49 colonies per ml of blood—an amount that would be considered extremely high and lethal in adult patients (31). As stated above, the most recent studies tend not to report the magnitude of bacterial burden in human patients with sepsis, but simply whether they were culture positive or negative; this precludes a full assessment of the relationship between bacterial load and mortality across the age groups. However, many animal models using CFU/ml blood as an outcome validate the observation that neonates are able to survive much higher circulating bacterial loads than adults (36, 39). Note that this is not to suggest that newborns are able to survive higher levels of bacterial exposure than adults (in fact, the opposite is true, as detailed below), rather that neonates are able to survive levels of bacteremia that adults cannot.

## The Balance of Disease Resistance vs. Immunopathology

Many studies have described deficiencies in the neonatal innate immune system that could be responsible for the decreased ability to clear invasive pathogens. For example, kinetics of pathogen clearance in animal models of neonatal infection show that neonates take longer to clear invasive bacteria than their adult counterparts (36, 39). A recent study comparing methicillin-resistant *Staphylococcus aureus* infection in neonatal and adult mice attributed a delayed clearance in neonates to inefficient phagocytosis and a limited neutrophil recruitment to the site of infection. Specifically, in neonates, neutrophil production dropped off despite the continued presence of bacteria, whereas in adult animals, a diminishing neutrophil production corresponded with bacterial clearance. Other studies have implicated impaired neutrophil recruitment as a potential explanation for the increased susceptibility to infection in early life (40, 41). Furthermore, while neonates have higher basal levels of circulating phagocytic cells than adults, they are generally considered to be less efficient phagocytes (40, 42–45). For example, *in vitro* neonatal monocytes and neutrophils in whole blood cultures have been shown to have an impaired phagocytic ability of *Escherichia coli* and *S. aureus* when compared to adults (44). However, other groups that found similarly reduced phagocytosis of *S. aureus* by newborn polymorphonuclear leukocytes (PMNs) also found that the exposure of neonatal PMNs to adult plasma resulted in adult levels of bactericidal activity and hydrogen peroxide production (against *S. aureus*) (17, 46). Similarly, phagocytosis of group B *Streptococci* and *E. coli* by adult and neonatal purified monocytes had similar phagocytic activity between the different age groups (45, 47). This brief excursion into the literature of just one aspect of host defense immediately highlights that the ability of newborn immune cells to fight infection is a purposeful response and not simply a state “deficient as compared to the adult.”

Just as *in vitro* comparisons of neonatal and adult phagocytic cells have contributed to the theory that neonatal susceptibility to infection is a result of “immaturity,” so has the evidence accrued which describes diminished *in vitro* pro-inflammatory responses when comparing neonatal and adult cells (7, 10, 48, 49). However, animal models of neonatal sepsis using a variety of pathogens (both bacterial or viral) or TLR agonists have found neonates to generate an inflammatory response equal to or greater than that of adults (1, 24, 39, 50–52). Furthermore, exogenous supplementations of pro-inflammatory cytokines have been shown to greatly increase mortality in a polymicrobial model of sepsis in neonatal mice (53, 54). This increased mortality of neonatal sepsis does not relate to a decreased bacterial clearance, as neonatal mice also suffer a much greater mortality than adults when challenged with purified TLR agonists in the absence of an infection (24, 26). This has led to the realization that the inflammatory response itself is considered to be largely responsible for the higher mortality of infected newborns vs. adults (53, 54).

Given this higher risk of the newborn vs. adult to suffer from the immune response to an infection (or TLR agonist), newborns would benefit from mechanisms that would reduce the risk to unleash a harmful antimicrobial immune response. The molecular mechanisms related to this have recently begun to be deciphered and highlight a direct connection to disease tolerance. An *E. coli* model of neonatal sepsis found that neonatal TRIF^−/−^ mice suffered a higher mortality than WT or MyD88^−/−^ strains with the opposite being true in young adults (55). Neonatal prioritization of TRIF-dependent pathway activation when exposed to TLR agonists was then linked to a strong induction of type 1 interferon regulatory responses, as opposed to the adult MyD88-dependent pro-inflammatory response. A molecular explanation for these age-dependent differences in defense strategy has recently been identified as the endogenous, heterodimeric complex of TLR4 ligands S100A8/A9: high levels of S100A8/A9 shift TLR signaling from MyD88- to TRIF-dependent pathways. S100A8/A9 alarmins are also known to be massively released at birth. This alarmin release is entirely incongruous with the “immune immaturity” paradigm as it represents a purposeful shift away from MyD88 pathway activation, the preferred adult pathway. If neonatal death was driven by a simple lack of adult-like features, one would expect that any external shift toward a more adult-like immune response would lead to better outcome. But the opposite is in fact the case, as *S100a9*^−/−^ neonatal mice suffer much higher mortality than their WT counterparts when infected, implying the alarmin release at birth; i.e., the subsequent shift away from an adult-like response is an important and necessary step to successfully mount a defense against an early-life infection (11, 56). The age-dependent production of S100A8/A9 thus represents an example of disease tolerance unique to neonates that has developed to avoid immunopathology from an MyD88-driven pro-inflammatory response at the potential cost of rapid bacterial clearance.

This emphasis on the TRIF-dependent response is entirely incongruous with the “immune immaturity” paradigm. While this is true for pathogens that signal through TLR4, other mechanisms of disease tolerance to, e.g., Gram-positive infections still need to be identified. For example, there are several other mechanisms in place in early life that commonly are described as immune suppressive, with the notion that these are remnants of the mechanisms that allow semiallogeneic mismatch *in utero* without rejection of maternal cells by the fetus (12). However, these mechanisms persist far beyond the immediate perinatal period and thus likely have other benefits in postnatal life, such as increasing disease tolerance by reducing immune-mediated pathology (“immune cost”) even if it comes at the cost of an increased bacterial burden (13).

## The Balance of Disease Resistance vs. Disease Tolerance

**Figure 1:**
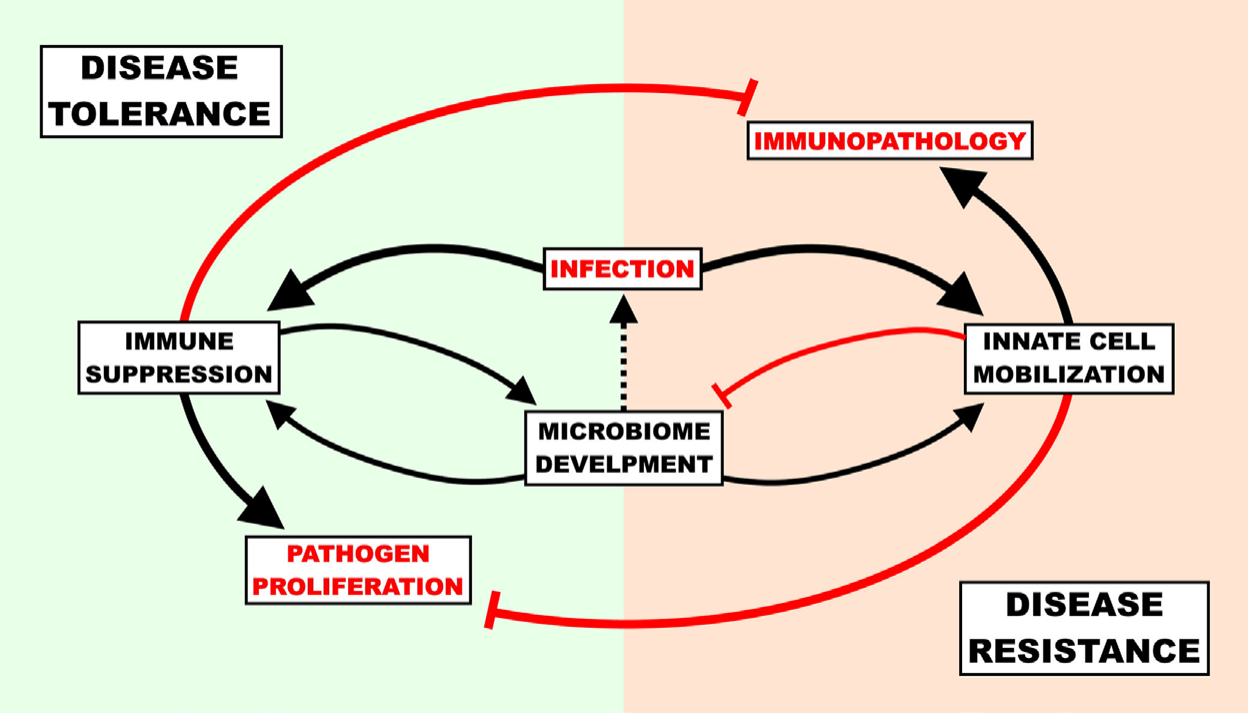
The cost of host-defense strategies in newborn infection. The immune response must be suppressed to a degree in order to allow healthy commensal colonization of the gut, though unchecked suppression can result in gut “leakiness” and lead to infection. Upon infection, newborns must balance the potential self-inflicted harm associated with the pro-inflammatory/antimicrobial response (immunopathology) with the dangers of unencumbered pathogen proliferation and ensuing virulence. A disease tolerance strategy reduces immunopathology and supports microbiome development at the cost of pathogen load, while a disease resistance strategy reduces pathogen load at the cost of microbiome development and immunopathology.

The benefit of disease tolerance as a host-defense strategy depends on the capacity for virulence of a given invasive agent. If the only pathogen ever encountered by a host organism secreted virulence factors that inflicted mortality in 100% of cases, there would be intense pressure to improve resistance and no pressure to improve tolerance. More relevant to humans is the opposite case; there are myriads of bacteria that rarely cause mortality and provide both direct and indirect fitness advantages to the host. This creates a situation where disease tolerance is a viable defense strategy, but to a finite degree. Even very low virulence organisms, if left totally unchecked, would cause disease. To prevent disease from occurring upon the transition from the semi-sterile environment *in utero* into the microbe-rich *ex utero* world, disease tolerance (immunosuppression preventing immunopathology) and disease resistance (inflammatory/antimicrobial responses preventing virulence) must maintain a state of homeostasis for optimal host defense. Without active suppression of inflammatory innate signaling, the initial influx of microbes from the birth process could prompt an enormous, potentially lethal inflammatory response; even if this inflammatory response did not result in mortality, there would be serious short- and long-term health ramifications as a result of inadequate bacterial diversity in the gut (25, 57). If there was no disease resistance, opportunistic colonizers would inevitably reach the blood stream and cause disease (Figure 1). Since adults (a) are less sensitive to immunopathology caused by inflammation, (b) have already established an enteric microbiome, and (c) are not hindered by the energetic requirements of development and environmental change (see below); the benefits of disease resistance (keeping pathogens out) outweigh the costs of disease tolerance (letting pathogens in).

## The Cost of Host Defense

**Figure 2:**
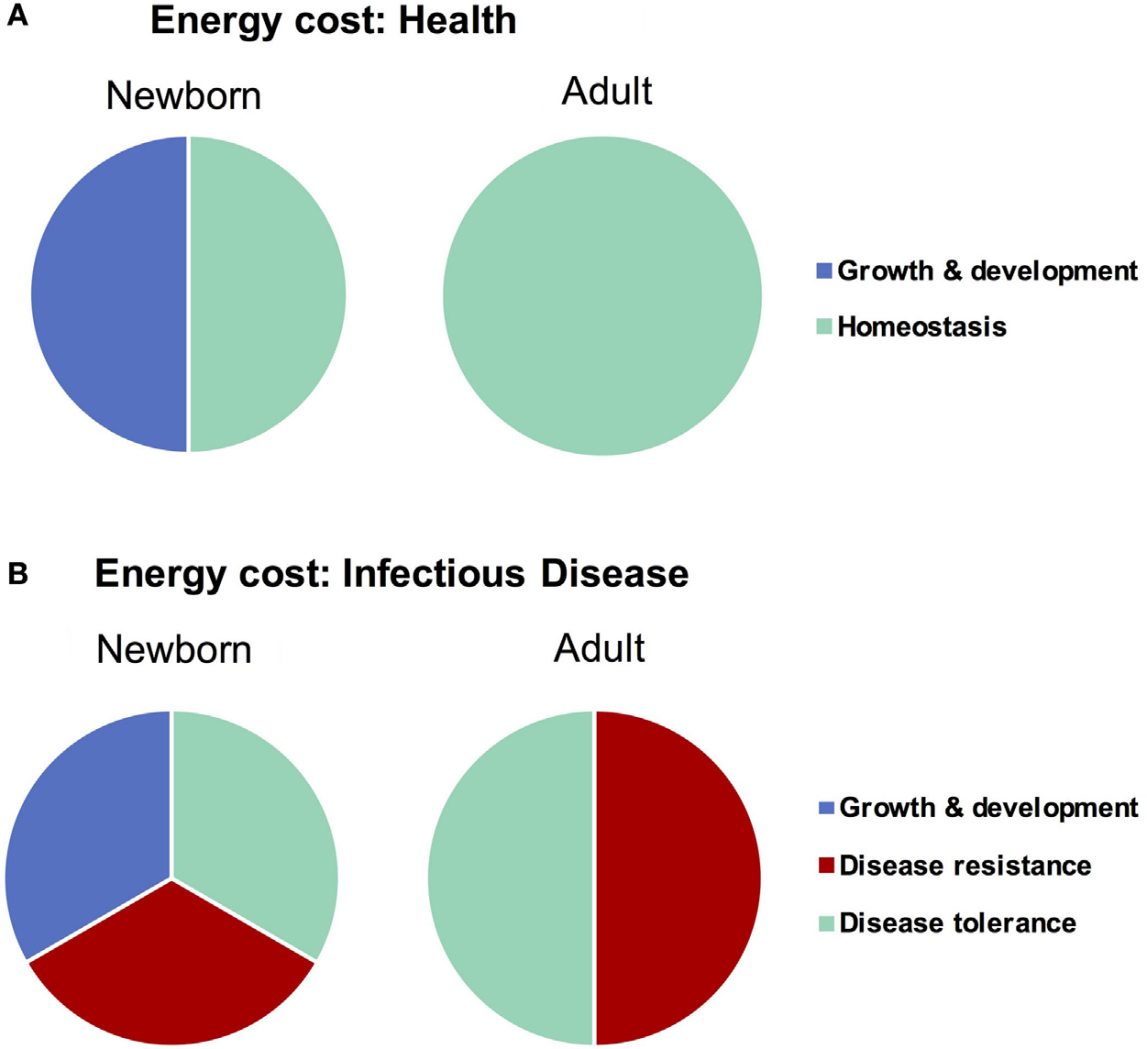
Difference in energy demands of the newborn and adult as it relates to infectious disease. Newborns must devote a large amount of energy toward growth and development which adults are able to spend on maintaining homeostasis. When healthy **(A)**, these differences in energetic demands may not be important, though when fighting infectious disease **(B)**, the newborn is unable to expend the resources required to employ a strategy of disease resistance and must therefore rely more heavily on disease tolerance.

Any form of host response (or lack thereof) to an invasive agent must be weighed in terms of the potential for self-inflicted damage, or immunopathology. The resultant immunopathological impact of any given response can range from negligible (i.e., mild fever) to fatal (i.e., septic shock), and thus the immunopathology associated with an immune response has been described as the “immune cost” of a response (58). The three principle host-defense strategies of avoidance, disease tolerance, and disease resistance can be ordered in terms of increasing immune cost, i.e., immune pathology: avoidance has a very low cost, resistance a very high cost, and tolerance lies somewhere in between (13). In addition to the cost of damage from an immune response, however, there is also an associated “energetic cost” which describes the amount of energy required to deploy a given strategy. Ordering the strategies by energetic cost indicates the same order as that of immune cost—disease avoidance very low (primarily behavioral, little to no regulation), disease resistance very high (58) (massive, highly regulated cell mobilizations across the body), and disease tolerance in the middle (some regulatory maintenance to avoid resistance and tissue healing). Both types of costs, immune and energetic, are particularly important to consider when discussing infections in neonates, as newborns are (a) particularly sensitive and prone to immunopathology (24) and (b) in the midst of a rapid growth and development phase which demands a high energy input to be maintained (59), i.e., neonates are unable to “pay” the costs of a full resistance response (Figure 2). Avoidance has failed by definition when discussing an already established active infection, which leaves disease tolerance as the primary defense strategy for newborns to cope with an invasive agent. This comprehensive, holistic point of view takes into account aspects of immunity (i.e., energy balance) which fall beyond the narrowly defined immune system and is best captured with the phrase “host fitness cost.” The concept of host fitness cost helps better explain some seemingly paradoxical observations in neonatal immunity and can inform interventions moving forward.

## The Role of the Microbiome in Neonatal Host Defense

In the last decade, a vast body of research has emerged, implicating the microbiome as a critical mediator of neonatal immune development (2, 6, 28, 60–62). Dysbiosis during the neonatal period has been associated with necrotizing enterocolitis, and both early- and late-onset sepsis (60, 63–65). Given the potential costs of impaired microbiome development, the neonatal immune system seems to have developed specific mechanisms to ensure “safe colonization” of the interphase between external and internal environments. Some of these mechanisms are reviewed below. The active immunosuppressive portion of the neonatal immune response may not only serve to minimize the damage associated with immunopathology but also ensure that the neonatal gut can be colonized with a large and diverse array of commensal bacteria. The interplay and dependence on commensal bacteria begins immediately with colonization, as transcriptomic analysis of germ-free mice exposed to common commensal bacteria showed the most prominent changes in genes associated with toll-like receptor (TLR) and type 1 interferon (IFN1) signaling pathways, which, as evidenced by the aforementioned importance of the TRIF-dependent signaling, are crucial in early life (66).

Newborns heavily depend on avoidance to prevent infection. In the context of microbial colonization, they rely on their mothers to introduce them to the right organisms and promote their growth, meanwhile avoiding unwanted colonizers from outcompeting the beneficial ones. While maternal influence on prepartum colonization is still being debated (67), postpartum colonization has been shown to be largely derived from the vagina during birth (68). In newborns sampled during the first week of life alongside their mothers, the majority of taxa detected in their stools were also detected in stool samples taken from their mothers at the same time (69). While newborns are colonized by bacterial families such as *Bacteroides* and *Clostridia* acquired from their mothers, the composition of their microbiota is still different from their mothers with *Escherichia*/*Shigella, Bifidobacterium, Streptococcus*, and *Enterococcus* occupying roughly half the space of the entire intestinal microbiome in newborns but only about 10% in their mothers [the exception is *Bacteroides*, which feature prominently in both (69)]. While life will eventually expose an individual to a multitude of different foods and diverse microbial environments, newborns subsist solely on breast milk and have no environmental exposure in their control. Thus, microbes found in human milk or other maternal sources that utilize human milk oligosaccharides dominate initial colonization (70, 71) and in turn provide resistance to colonization by potential pathogens (72) and other forms of immune support (28). Consequences of less-controlled exposure are suggested by the detriment of deviation of exclusive breastfeeding practices; excusive breastfeeding in low- and middle-income countries (and perhaps high income) is associated with a substantial reduction in newborn disease and mortality (18).

Avoiding inflammatory or deleterious responses to commensal microbes is important throughout the life span of colonized hosts. Strategies put in place by the newborn are appropriate for the environmental pressures and physiological requirements of this unique early-life period. Adults depend on a thick mucous layer packed with antimicrobial peptides and dimerized IgA alongside trained innate and adaptive mucosal responses to prevent microbial translocation into host tissues (73). Since the development of these defenses first requires stimulation by the microbiota, newborns must employ a different repertoire of tolerance strategies prior to the introduction of solid food. For example, newborn intestinal epithelial cells (IECs) produce a micro-RNA molecule that targets IRAK-1, a necessary signal transducer of inflammatory TLR signaling for degradation and thus reduces inflammation caused by commensal stimulation of intestinal TLRs (74). However, this mechanism requires continuous TLR4 stimulation for its maintenance and is absent in pups delivered by C-section. Also, murine IECs produce antimicrobial peptide CRAMP only prior to Paneth cell development and show some efficacy against *Listeria* infection (75). In fact, weaning appears to be a massive transitory period for IEC regulation. Transcriptional regulator Blimp1 is active in the newborn intestine and ceases to be expressed upon weaning; its deletion results in an adult-like intestinal architecture at birth and with it a substantial early-life mortality in animal models (76). A more comprehensive evaluation of intestinal transcriptional regulation showed a more global postweaning shift in rodents with an increase in IL-1/TLR signaling post weaning that was lost in MyD88/TRIF^−/−^ mice, showing that the intestinal immune environment is very sensitive to changes in early-life transitions (77) and is likely guided by the changing microbial and nutritional environment.

Newborn colonizers also play an instrumental role in preventing immune hyperresponsiveness within and outside the mucosal immune system. Widely studied commensal *Bacteroides fragilis* promotes an anti-inflammatory environment by inhibiting the recruitment of invariant NKT cells to the gut and lung mucosa, leaving mice less susceptible to inflammatory disease later in life (78). dsRNA from lactic acid bacteria (LAB) preferentially promotes IFN-β expression in mucosal dendritic cells to concentrations that predominantly drive their anti-inflammatory effects in adult rodents (79). Since LAB are prominent colonizers of the newborn gut, it is likely that they perform similar functions during this period—although that mechanism still needs to be investigated. An influx of highly activated regulatory T cells into the neonatal skin has been linked with tolerance to commensal skin bacteria, an event that was not replicated when the same experiment was performed in adult animals. Selective inhibition of these specific Tregs completely prevented tolerance to commensal bacteria colonization later in life (80).

There is evidence in adult animal models that microbiome-derived products can reduce disease pathology, i.e., increase disease tolerance. Recently, the clostridia-derived metabolite desaminotyrosine (DAT) was shown to promote type 1 IFN signaling in lung dendritic cells, resulting in a less damaging response to influenza challenge and an increased animal survival, while the viral burden in DAT-treated mice remained unchanged (81). Newborns are thought to be at risk for “inside-out” infections, where the pathogen escapes mucosal compartments, supported by the identification of the same strain of bacteria from septic newborns in their feces (82). The microbiome is not only instrumental in excluding potential pathogens, but by promoting an anti-inflammatory environment, it likely also plays a role in reducing the potential harm from responses to inflammatory microbes. For example, newborn mice given probiotic strains of *Lactobacillus* were rescued from death caused by *Citrobacter rodentium* infection (a mouse model of enteropathogenic *E. coli*) *via* a mechanism involving the recruitment of Tregs to the colon (83). A second group was administered *L. acidophilus* alongside a prebiotic to newborn mice prior to challenge with *C. rodentium* in young adulthood, a finding that treated mice had an enhanced IL-10 and a diminished NF-kB response to infection, in addition to a faster recovery from disease (84).

Taken together, host-commensal bacteria crosstalk in newborns is highly dependent on maternal care for both original inoculation and continued support through breastfeeding and controlled environmental exposure. This complete dependence is unique to the newborn period, highlighted by both the devastating consequences of suboptimal breastfeeding practices and the reworking of intestinal and microbial architecture once solid foods are introduced. During this time, commensals selected to thrive in newborns promote disease tolerance by boosting anti-inflammatory immune responses and disease avoidance by excluding potentially pathogenic organisms from coming into contact with the epithelium, thus preventing infection of the mucosal and systemic sites.

## Translational Impact

Successful immune defense relies on a balance between disease resistance and disease tolerance strategies to bring the host back to homeostasis. The ideal intervention is one that would hasten restoration of homeostasis or enable the system to deal with an extreme imbalance in either direction for longer periods. A promising approach that fits this requirement is to work in the realm of innate immune memory or trained immunity—the concept that an initial infection or an exposure to a pathogen can provoke an enhanced innate immune response when the organism is re-exposed or exposed to a different pathogen (85) Unlike many traditional interventions, prophylactic or treatment approaches reliant on trained immunity are not dependent on shifting the response only toward resistance. Numerous examples of successful interventions reliant on innate immune memory have been described in animal models and human clinical trials (85). Various TLR agonists have, for example, shown to protect nonspecifically against mortality from a polymicrobial challenge 24 h later in neonatal mice (41). A similar model used a *Listeria monocytogenes* challenge and found TLR agonists to be protective as well. Cord blood monocytes stimulated with endotoxin showed an enhanced activity both 7 and 14 days later. Moreover, a retrospective analysis revealed correlation between histological chorioamnionitis (a condition prompting an inflammatory response) exposure and a reduction in late-onset neonatal sepsis (85). Most impressively, probiotics in newborns have been shown to be very powerful in reducing both necrotizing enterocolitis (86), a devastating disease characterized by colonization with proteobacteria and excessive inflammation (87), and most recently, sepsis and respiratory disease when administered within days of birth (88). Finally, certain live vaccines (particularly Bacille Calmette–Guérin) have been shown to reduce all-cause neonatal mortality, presumably through nonspecific protection against unrelated pathogens in the first month of life (89).

## Summary

The paradigm that neonates are more susceptible to infectious disease than adults is well known, well documented, yet poorly understood. The high susceptibility and mortality figures have largely been attributed to “immune immaturity,” a vague concept that is predicated on findings of weaker antimicrobial responses of newborns than those of adults. Here, we posit that an increased susceptibility to infection in neonates is not a result of immaturity but rather one of immunosuppressions, which is in part an active defense strategy termed disease tolerance. This is supported by the finding that neonates can survive significantly higher bacterial loads than adults during active infection. This observation is consistent across many studies, yet still is oft ignored and cannot be adequately explained by the immaturity paradigm. Employing a defense strategy of disease tolerance during infection rather than disease resistance confers some advantages but is more likely a virtue of necessity. Compared to the adult-like disease resistance strategy, disease tolerance is (a) less energetically intensive (critical during a period of rapid development), (b) less likely to incur serious damage associated with bacterial clearance (neonates are more sensitive to immunopathology than adults and seem to have a heightened innate immune response), and (c) less likely to interfere with the development of the gut microbiome (overactive resistance pathways could result in a dangerous inflammatory response and interfere with colonization). Maintaining homeostasis between disease resistance and disease tolerance is a critical outcome of fighting and preventing infection. Fortunately, interventions, which work within these constraints, have been identified and promise to finally usher in the desperately needed reduction of global newborn mortality rates.

## Author Contributions

DH, NA, and TK conceived of the presented ideas. DH wrote the manuscript with assistance from RB-O, NH, and TK. All authors provided critical feedback and editing through the writing process.

## Conflict of Interest Statement

The authors declare that the research was conducted in the absence of any commercial or financial relationships that could be construed as a potential conflict of interest.
